# Allicin Alleviates Dextran Sodium Sulfate- (DSS-) Induced Ulcerative Colitis in BALB/c Mice

**DOI:** 10.1155/2015/605208

**Published:** 2015-05-05

**Authors:** Ashok Kumar Pandurangan, Salmiah Ismail, Zeinab Saadatdoust, Norhaizan Mohd. Esa

**Affiliations:** ^1^Department of Nutrition and Dietetics, Faculty of Medicine and Health Sciences, Universiti Putra Malaysia, 43400 Serdang, Selangor, Malaysia; ^2^Department of Pharmacology, Faculty of Medicine, University of Malaya, 50603 Kuala Lumpur, Malaysia; ^3^Laboratory of Molecular Biomedicine, Institute of Bioscience, Universiti Putra Malaysia, 43400 Serdang, Selangor, Malaysia

## Abstract

The objective of this study is to evaluate the effect of allicin (10 mg/kg body weight, orally) in an experimental murine model of UC by administering 2.5% dextran sodium sulfate (DSS) in drinking water to BALB/c mice. DSS-induced mice presented reduced body weight, which was improved by allicin administration. We noted increases in CD68 expression, myeloperoxidase (MPO) activities, and Malonaldehyde (MDA) and mRNA levels of proinflammatory cytokines, such as* tumor necrosis factor- (TNF-) α, interleukin- (IL-) 1β, IL-6*, and* IL-17*, and decrease in the activities of enzymic antioxidants such as superoxide dismutase (SOD), Catalase (CAT), Glutathione reductase (GR), and Glutathione peroxidase (GPx) in DSS-induced mice. However, allicin treatment significantly decreased CD68, MPO, MDA, and proinflammatory cytokines and increased the enzymic antioxidants significantly (*P* < 0.05). In addition, allicin was capable of reducing the activation and nuclear accumulation of signal transducer and activator of transcription 3 (STAT3), thereby preventing degradation of the inhibitory protein I*κ*B and inducing inhibition of the nuclear translocation of nuclear factor (NF)-*κ*B-p65 in the colonic mucosa. These findings suggest that allicin exerts clinically useful anti-inflammatory effects mediated through the suppression of the NF-*κ*B and IL-6/p-STAT3^Y705^ pathways.

## 1. Introduction

Ulcerative colitis (UC) and Crohn's disease (CD), which are considered inflammatory bowel diseases (IBDs), are chronic gastrointestinal disorders characterized by inflammation in the intestine and colon, as well as mucosal tissue damage. IBD is a multifactorial disease with unknown etiology [1]. However, it is believed that the pathophysiology of IBD involves an interaction among environmental, genetic, and immunological factors. Chronic inflammation in the intestine may be due to activation of the immune system by normal intestinal bacteria. The main clinical manifestations are abdominal pain, diarrhea, mucous, bloody, and purulent stools, recurrent attacks, and relapse [2]. The activation of the immune system results in the production of proinflammatory cytokines (TNF-*α*, IL-1*β*, IL-6, IL-17, and IL-21), reactive oxygen metabolites (ROM), and prostaglandins [3, 4]. These mediators have been suggested to contribute to the development of mucosal damage of the colon and lead to a chronic inflammatory process.

Nuclear factor- (NF-) *κ*B is a vital transcription factor that has the ability to induce the expression of a large array of inflammatory mediators and plays a role as a core transcription factor in diverse immune responses, and NF-*κ*B signaling has been recognized as a major pathway responsible for UC [5]. IL-6 is a key NF-*κ*B-dependent cytokine that binds to a soluble form of the IL-6 receptor (sIL-6R), and these complexes interact with gp130, which then dimerizes to induce the activation of signal transducer and activator of transcription 3 (STAT3). STAT3 is a cytoplasmic protein that functions as a transcriptional activator and plays a pivotal role in the regulation of different types of immune and inflammatory responses. The activation of STAT3 involves the process of phosphorylation at tyrosine residues. Phosphorylated STAT3 dimerizes and translocates to the nucleus, where it binds to specific DNA motifs and activates the transcription of distinct groups of genes. STAT3 signaling is involved in the pathogenesis of UC and is known to be a target in the treatment of UC and CAC [6, 7].

Allicin, a sulfur-containing natural compound with many different biological properties, is responsible for the typical smell and taste of freshly cut or crushed garlic [8]. Allicin has many beneficial effects, including antioxidant, anti-inflammatory [9], antiproliferative [10], and proapoptotic effects [11]. Allicin protects rat cardiomyoblasts (H9c2 cells) from hydrogen peroxide-induced oxidative injury through inhibiting the generation of intracellular reactive oxygen species (ROS) [12]. Allicin is also known to reduce acrylamide-induced toxicity in mice [13]. In the present investigation, we studied the anti-inflammatory activity of allicin against dextran sodium sulfate- (DSS-) induced colitis in BALB/c mice and the possible underlying molecular mechanisms.

## 2. Materials and Methods

### 2.1. Extraction of Allicin

Allicin was extracted from fresh garlic cloves through two steps using the methods described by Bat-Chen et al. [14]. First, 10 g of fresh garlic cloves was peeled and crushed with 100 mL of water and 10 g of NaCl for 5 min using a glass blender. The mixture was then poured into a separating funnel, and 100 mL of diethyl ether was added; the solution was mixed vigorously and allowed to stand for 15 min until two distinct phases appeared. The organic phase was evaporated under reduced pressure (Buchi Rotavapor, Flawil, Switzerland) at room temperature. The residue was washed with double-distilled water filtered using Whatman No. 1 paper. The amount of water used determined the final concentration of allicin, and the purified allicin solution was stored at 4°C.

### 2.2. Animals and Experimental Design

BALB/c mice weighing 25–30 g were purchased from A Sapphire Enterprise (43300 Serdang, Selangor, Malaysia). The animals were housed in plastic cages with woodchip bedding in a well-ventilated and temperature-controlled (25–27°C) room with a 12-h light/12-h dark cycle. After one week of acclimation, the mice were randomly sorted into three different experimental groups and were provided food and water* ad libitum*. After acclimatization, the mice in groups 2 and 3 were administered 2.5% DSS (MP Biochemicals, OH, USA) in drinking water for seven days. The mice in group 3 were orally treated with allicin at the dose of 10 mg/kg body weight. The body weights were monitored every day, and the food consumption was recorded throughout the experimental period.

The mice were sacrificed on day 8, and the colons (from the ileocecal junction to the anal verge) were removed. After measurement of the lengths and weights, the colons were cut open longitudinally along the main axis and washed with phosphate-buffered saline (PBS; pH 7.4). After gross examination, the colons were fixed in 10% neutral-buffered formalin for histological and immunohistochemical analyses. The remaining colons were used for mRNA and western blot analyses.

### 2.3. Histological Analysis

Paraffin-embedded samples were cut into 5 micrometer sections and then stained with hematoxylin and eosin (HE) for light microscopic examination. The sections of the colon were photographed with a Nikon ECLIPSE 80i (Tokyo, Japan) photomicroscope. The samples were analyzed and scored as described previously [15, 16].

### 2.4. Measurement of Myeloperoxidase Activity

The activity of myeloperoxidase (MPO) in the colon homogenates was determined as previously described with some modifications [17]. Briefly, the whole-colon tissue was homogenized at a concentration of 0.1 g/mL in PBS containing a complete protease inhibitor cocktail and centrifuged at 12,000 g for 10 min. The pellets were resuspended in 50 mM sodium phosphate, pH 6.0, containing 0.5% hexadecyltrimethylammonium bromide, crushed on ice, and subjected to three freeze/thaw cycles. The lysate was centrifuged at 14,000 g for 10 min, and the supernatant was heated at 60°C for 2 h to inhibit any catalase activity. The sample was added to the reagent buffer (50 mM sodium phosphate, pH 6.0, 0.8 mM 3,3′,5,5′-tetramethylbenzidine, and 5 mM hydrogen peroxide). The MPO-dependent oxidation of 3,3′,5,5′-tetramethylbenzidine was measured by monitoring the absorbance at 650 nm. The protein concentrations were determined using the BCA protein assay (Nacalai Tesque, Tokyo, Japan).

### 2.5. Malondialdehyde (MDA) Measurement

The levels of malondialdehyde in the colon were determined as an indicator of lipid peroxidation was estimated by the method of Ohkawa et al. [18]. Colon tissue was homogenized in 1.15% KCl solution. The sample consisted of 0.2 mL 8.1% SDS, 1.5 mL 20% acetic acid, 1.5 mL 0.8% thiobarbituric acid, and 0.7 mL distilled water. Samples were boiled for 1 h at 95°C and centrifuged at 3000 ×g for 10 min. The absorbance of the supernatant was measured spectrophotometrically at 650 nm.

### 2.6. Assay of Colonic Enzymic Antioxidants

The assay of superoxide dismutase (SOD) was followed by the method of Kakkar et al. [19], based on 50% inhibition of the formation of NADH-phenazine methosulphate-nitroblue tetrazolium (NBT) formazan at 520 nm. One unit of the enzyme activity was taken as the amount of enzyme required for 50% inhibition of NBT reduction/minute/mg protein. The activity of catalase (CAT) was determined by the method of Sinha [20]. The values of CAT activity are expressed as micromole of H_2_O_2_ utilized/minute/milligram protein. Glutathione reductase (GR) activity was assayed using the method of Carlberg and Mannervik, [21] and the values are expressed as *μ*mol of NADPH oxidized/minute/mg protein. Glutathione peroxidase (GPx) activity was assayed by the method of Rotruck et al. [22] and the values are expressed as micromole of GSH utilized/minute/mg protein.

### 2.7. Confocal Microscopic Analysis

Paraffin-embedded colonic tissue sections with a thickness of 5 *μ*m were deparaffinized in xylene and then rehydrated in a graded series of ethanol solutions. The slides were then blocked with 5% BSA in TBS for 90 min. The sections were then immunostained with rabbit anti-CD68 antibody (NOVUS Biologicals, Littleton, CO, USA) diluted 1 : 100 with 5% BSA in TBS and incubated overnight at 4°C. After the sections were washed three times with TBS, the slides were then incubated with goat and rabbit DyLight 550 secondary antibody (Thermo Scientific, Rockford, IL, USA) diluted 1 : 200 with TBS and incubated in the dark for 120 min at room temperature. The sections were then washed with TBS and incubated with the nucleus-specific counterstain propidium iodide (Nacalai Tesque, Japan) or 4′,6-diamidino-2-phenylindole (DAPI; Invitrogen, Carlsbad, CA, USA) to stain the cell nuclei. The slides were mounted in an Ultracruz hard-set mounting medium (Santa Cruz Biotechnology Inc., Dallas, TX, USA), coverslipped, and visualized under a FV1200 laser-scanning confocal microscope (Olympus, Tokyo, Japan).

### 2.8. Immunofluorescence Analysis

The immunofluorescence method used in this study was described by Pandurangan et al. [23]. Paraffin-embedded colonic tissue sections with a thickness of 5 *μ*m were deparaffinized in xylene and then rehydrated in a graded series of ethanol solutions. The slides were then blocked with 5% BSA in TBS for 90 min. The sections were then immunostained with rabbit anti-NF-*κ*B (Santa Cruz Biotechnology, CA, USA) and anti-pSTAT3^Y705^ antibody (Cell Signaling Technology, CA, USA) diluted 1 : 100 with 5% BSA in TBS and incubated overnight at 4°C. After the sections were washed three times with TBS, the slides were incubated with goat and rabbit DyLight 550 secondary antibody (Thermo Scientific, Rockford, IL, USA) diluted 1 : 200 with TBS and incubated in the dark for 120 min at room temperature. The sections were then washed with TBS and incubated with the nucleus-specific counterstain propidium iodide (Nacalai Tesque, Japan) or 4′,6-diamidino-2-phenylindole (DAPI; Invitrogen, Carlsbad, CA, USA) to stain the cell nuclei. The slides were mounted in an Ultracruz hard-set mounting medium (Santa Cruz Biotechnology Inc., Dallas, TX, USA), coverslipped, and visualized under a FSX100 fluorescent microscope (Olympus, Tokyo, Japan).

### 2.9. Protein Extraction and Western Blot Analysis

Western blot analysis was performed according to the method described by Pandurangan et al. [24]. Briefly, colonic tissues were removed and washed in PBS. The whole tissue was cut into pieces and homogenized in five volumes of ice-cold homogenizing buffer (0.1 mM NaCl, 0.1 M Tris Cl, and 0.001 M EDTA) containing 1 mM PMSF, 1 mg/mL aprotinin, and 0.1 mM leupeptin at 3000 g and 4°C for 1 h. The protein content of the supernatants was estimated using BSA as a standard. The extracts were heated in a boiling water bath for 5 min, and the protein samples (40 *μ*g of each sample) were subjected to SDS-PAGE and transferred to PVDF membranes using a transfer apparatus (BioRad, USA). The membranes were blocked overnight at 4°C with blocking reagent (20 mM Tris (pH 7.4), 125 mM NaCl, 0.2% (v/v) Tween 20, 4% (w/v) nonfat dry milk, and 0.1% (w/v) sodium azide), incubated with p-STAT3^Y705^, T-STAT3 (Cell Signaling Technology, USA), NF-*κ*B, I*κ*B, Bcl-xl, and *β*-actin (Santa Cruz Biotechnology, CA, USA) primary antibodies at the appropriate dilutions recommended by the supplier for 2 h, and incubated with the corresponding horseradish peroxidase- (HRP-) conjugated secondary antibody (Santa Cruz Biotech, CA, USA) for 1 h. The protein-antibody complexes were detected using the Clarity western ECL substrate (BioRad, USA), and the results were quantified using the ImageJ software (NIH, Bethesda, MD, USA).

### 2.10. RNA Isolation and Quantitative PCR

The tissue samples were frozen and mechanically dissociated in RNA buffer. The total RNA was then extracted using the QIAshredder and RNeasy Kit (QIAGEN, Hilden, Germany) following the manufacturer's instructions. Real-time PCR was performed using an Eppendorf PCR system with the QuantiFast SYBR Green PCR Master Mix (QIAGEN, Hilden, Germany), primers (1 mM; Table 1), and 1 *μ*g of cDNA in a 25-mL reaction mixture. Each target and standard *β*-actin cDNA were analyzed in duplicate through three independent real-time RT-PCR assays. Thermal cycling was initiated with an activation step of 30 s at 95°C, and this step was followed by 40 cycles of 95°C for 5 s and 60°C for 30 s. Immediately after amplification, melt curve protocols were performed to ensure that primer dimers and other nonspecific products were minimized. The relative expression of the target genes was analyzed by the ΔΔCt method.

### 2.11. Statistical Analysis

The data are expressed as the means ± S.D. The data were processed using the SPSS version 16.0 statistical analysis software (SPSS Inc., Chicago, IL, USA). All *P* values were two-tailed, and a *P* value of less than 0.05 was considered significant.

## 3. Results

### 3.1. General Observations

**Figure 1:**
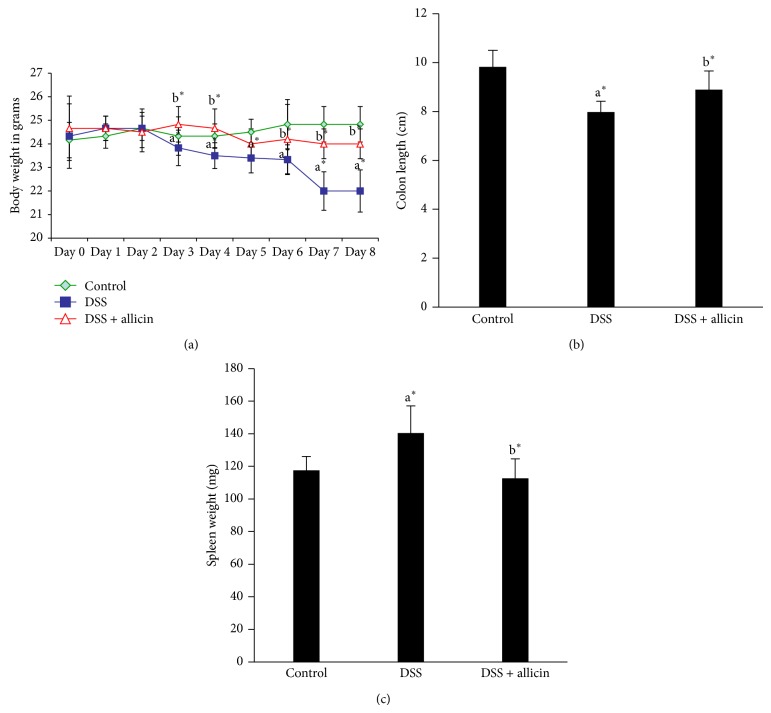
Allicin treatment improved DSS-induced colitis in mice. (a–c) Mice were administered 2.5% DSS in drinking water for seven days, and the next day, the mice were sacrificed. Allicin was administered orally from days 1 to 7. (a) The body weight loss observed in the DSS group was improved by allicin treatment. (b) Colon length. (c) Spleen weight. The values are expressed as the means ± S.D. In comparison of ^a^control versus DSS, ^b^DSS versus DSS + allicin. “∗” denotes a statistically significant difference at *P* < 0.05, and ns indicates a nonsignificant difference.

The consumption of food and water was measured throughout the experiment, and there were no significant differences between the groups (data not shown). The oral administration of DSS for seven days induced acute colitis characterized by loss in body weight in mice starting on day 3 compared with the control mice (Figure 1(a)). Statistically significant differences (*P* < 0.05) were obtained starting on day 4. However, the administration of allicin improved the body weight, and statistically significant differences (*P* < 0.05) were obtained starting on day 5. It has been reported that the colon length is inversely associated with the severity of DSS-induced colitis [25]. Significant shortening of the colon length was observed in the DSS-induced mice, and this decrease was suppressed by treatment with allicin (Figure 1(b)). The spleen weights of the mice administered DSS were significantly (*P* < 0.05) higher compared with those of the control group, and allicin treatment significantly decreased the spleen weights (*P* < 0.05; Figure 1(c)).

### 3.2. Allicin Reduces Microscopic Colon Damage in DSS-Induced Acute Colitis

**Figure 2:**
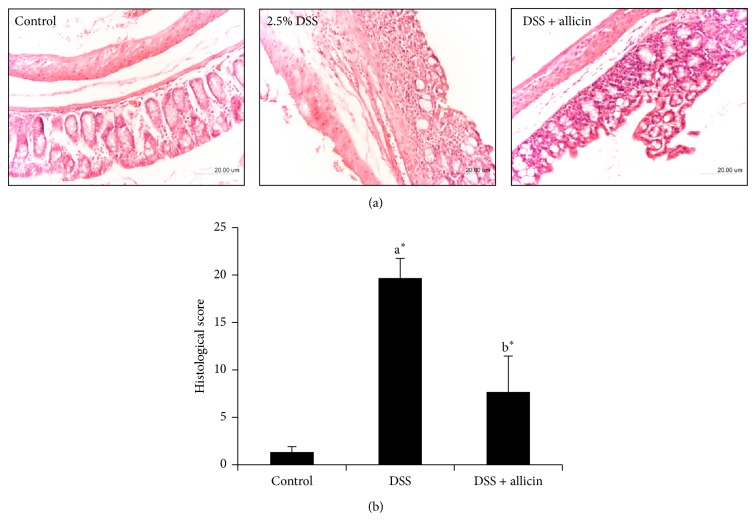
Allicin treatment prevented DSS-induced colon damage in mice. (a) Sections of colon tissues were stained with H&E. (b) The histopathological scores for each group were determined. The values are expressed as the means ± S.D. Comparisons: ^a^control versus DSS, ^b^DSS versus DSS + allicin. “∗” denotes a statistically significant difference at *P* < 0.05, and ns indicates a nonsignificant difference.

The histological and morphological characteristics of the colons were assessed after H&E staining, and representative results as well as the microscopic scores are shown in Figures 2(a) and 2(b). In the control mice (vehicle group), the colons presented a normal morphology of crypts, abundant goblet cells, a small number of lamina propria mononuclear cells, no signs of mucosal thickening, and complete absence of ulcerations. However, the DSS-induced mice presented severe epithelial damage with extensive cellular infiltration into the lamina propria and colon mucosa, depletion of the goblet cells, mucosa thickening, and complete destruction of the architecture, resulting in a high microscopic damage score (Figure 2(b)). In contrast, treatment with allicin (10 mg/kg body weight) completely blocked inflammatory cell infiltration with minimal loss of epithelial cells, which resulted in a very low microscopic damage score, compared with the colons from mice treated with DSS.

### 3.3. Allicin Reduces the Expression/Activity of CD68 and MPO

**Figure 3:**
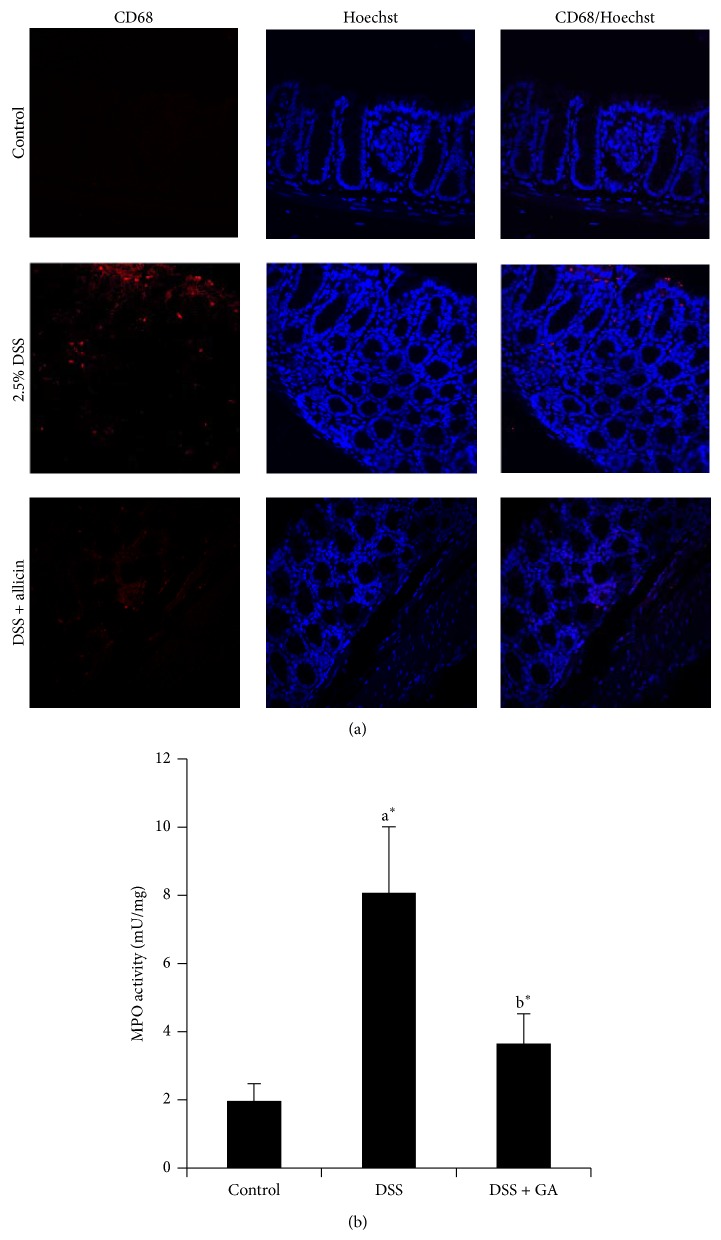
Allicin reduces the expression/activity of CD68 and MPO. (a) Confocal microscopic analysis of CD68 in the control and experimental animals. The experimental details are described in Materials and Methods. The secondary antibody used in this study was tagged with DyLight 550, and the cells were counterstained with Hoechst. The control animals exhibited lower CD68 expression. DSS treatment increased the expression of CD68 (Dylight 550; red). However, allicin treatment decreased the expression of CD68. (b) Activity of MPO. The MPO activity is presented as mU/mg of protein. The values are expressed as the means ± S.D. In comparison of  ^a^control versus DSS, ^b^DSS versus DSS + allicin. “∗” denotes a statistically significant difference at *P* < 0.05, and ns indicates not significant.

Figure 3(a) shows the confocal microscopic expression of CD68, a marker of macrophages/monocytes in the control and experimental mice. The administration of DSS resulted in increased expression of CD68 compared with the control. However, treatment with allicin reduced the expression of CD68.  Figure 3(b) presents the activity of MPO in the control and experimental mice. MPO is an enzyme present in neutrophils and at a much lower concentration in monocytes and macrophages. The level of MPO activity is directly proportional to the neutrophil concentration in the inflamed tissue. In addition, increased MPO activity has been reported to be an index of neutrophil infiltration and inflammation [26]. The oral administration of DSS leads to increased MPO activity compared with the control mice. In contrast, allicin treatment reduced the activity of MPO.

### 3.4. Allicin Reduces the mRNA Levels of Proinflammatory Cytokines

**Figure 4:**
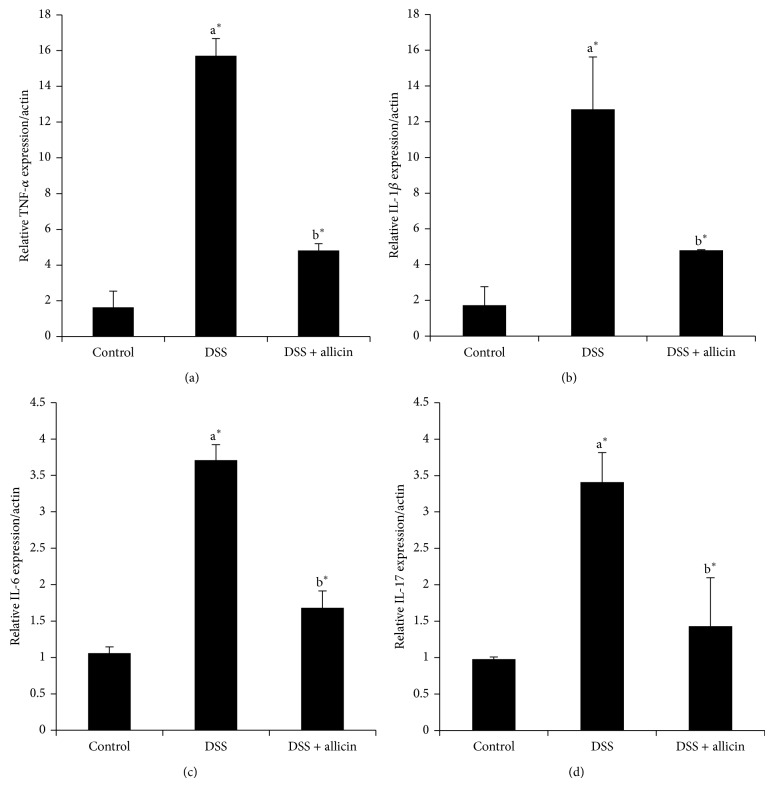
Allicin suppressed the expression of proinflammatory cytokines in colon tissues from DSS-colitis mice. The mRNA expression levels of the inflammation-related cytokines (a)* TNF*-*α*, (b)* IL-1β*, (c)* IL-6*, and (d)* IL-17A* in colonic tissues were determined by real-time PCR. The values are expressed as the means ± S.D. In comparison of  ^a^control versus DSS,  ^b^DSS versus DSS + allicin. “∗” denotes a statistically significant difference at *P* < 0.05, and ns indicates not significant.

Figure 4 depicts the qRT-PCR-measured expression levels of proinflammatory cytokines, such as* TNF-α* (Figure 4(a)),* IL-1β* (Figure 4(b)),* IL-6* (Figure 4(c)), and* IL-17* (Figure 4(d)), in the control and experimental mice. Many studies have shown that proinflammatory cytokines are elevated during UC [4]. Consistent with previous findings, we detected increased expression levels of cytokines compared with the control. However, the oral administration of allicin significantly decreased the expression of cytokines (*P* < 0.05).

### 3.5. Allicin Attenuates Level of MDA in AOM/DSS-Induced CAC

**Figure 5:**
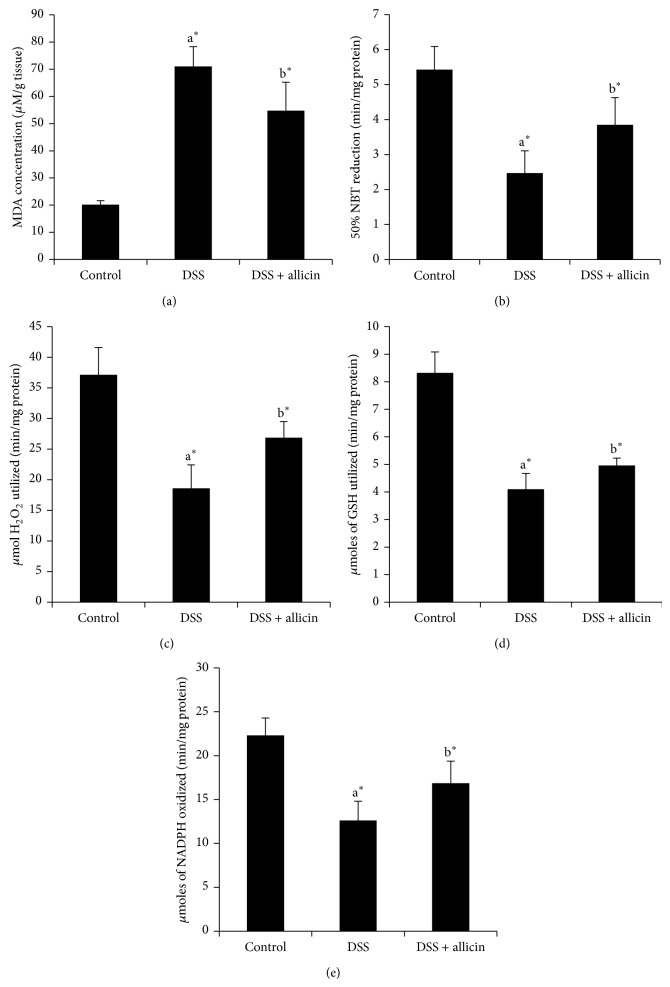
Allicin reduced the level of colonic MDA and increased the activity of colonic enzymic antioxidants in DSS-induced UC. (a) Levels of MDA, (b) activity of SOD, (c) activity of CAT, (d) activity of GPx, and (e) activity of GR. Values are expressed as mean ± S.D. for 6 mice in each group. In comparison of  ^a^control versus DSS, ^b^DSS versus DSS + allicin. “∗” denotes a statistically significant difference at *P* < 0.05, and ns indicates not significant.

MDA is an end product of lipid peroxidation that is considered as harmful and may be responsible for release of cell contents and cell death, causing tissue and organ damage [27].  Figure 5(a) showed the significant (*P* < 0.05) increase in the level of MDA in DSS-induced mice. We observed a significant (*P* < 0.05) reduction in the levels of MDA upon treatment with allicin.

### 3.6. Cocoa Increased the Activities of Enzymic Antioxidants in DSS-Induced UC

Effect of allicin on enzymic antioxidants such as SOD, CAT, GPx, and GR was shown in Figure 5. Our results indicate that the activities of SOD, CAT, GPx, and GR were significantly decreased (*P* < 0.05) in the colon tissues of DSS-induced mice as compared to the control mice. On the other hand supplementing allicin for seven days significantly (*P* < 0.05) elevated the SOD, CAT, GPx, and GR (*P* < 0.05) activities as compared to the mice induced with DSS.

### 3.7. Allicin Reduces the Expression of NF-*κ*B

**Figure 6:**
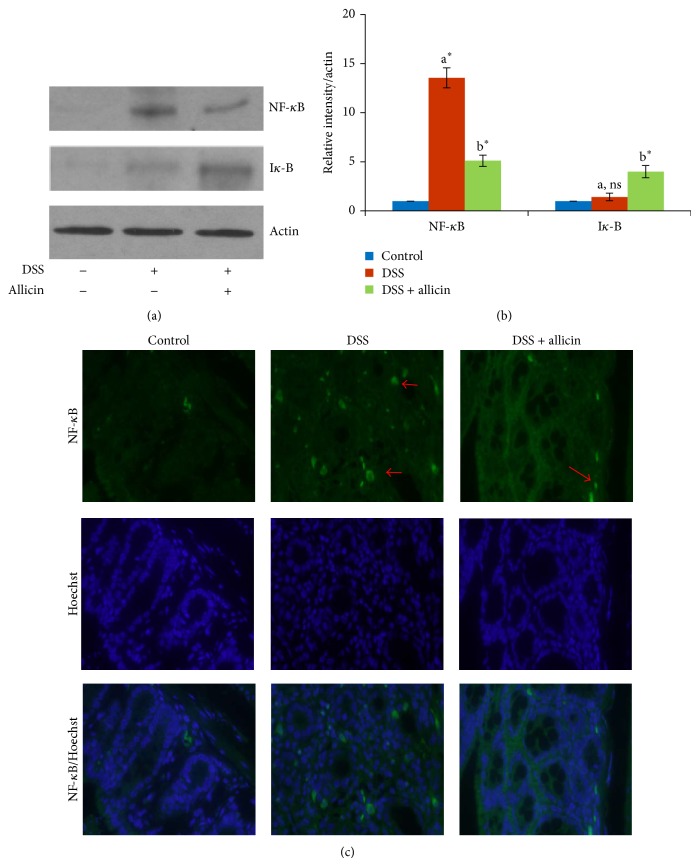
Allicin suppressed the expression of NF-*κ*B. (a) Western blot analysis of NF-*κ*B and I*κ*B. Allicin reduced the expression of NF-*κ*B. The DSS-treated (lane 2) mice showed increased expression levels of NF-*κ*B, and this increase was reduced by the subsequent administration of allicin (lane 3). (b) Densitometric quantification of blots using the ImageJ software (NIH, USA). The values are expressed as the means ± S.D. In comparison of  ^a^control versus DSS,  ^b^DSS versus DSS + allicin. “∗” denotes a statistically significant difference at *P* < 0.05, and ns indicates not significant. (c) An immunofluorescence analysis revealed strong expression of the activated form of NF-*κ*B (DSS group) and undetectable expression of NF-*κ*B in the normal colonic mucosa (control group). In comparison, the treatment of DSS-induced mice with allicin resulted in reduced expression of NF-*κ*B in the colon (20x).

Figure 5(a) shows the NF-*κ*B and I*κ*B levels in control and experimental animals detected through immunoblot analysis. NF-*κ*B is considered a good target for the treatment of diseases with an inflammatory component. Many naturally derived drugs attenuate the* in vivo* activation of this transcription factor by preventing I*κ*B phosphorylation and degradation [28]. We found an increased expression of NF-*κ*B in DSS-induced mice and markedly altered I*κ*B expression. However, allicin treatment decreased the expression of NF-*κ*B and increased the expression of I*κ*B. The quantification of the immunoblots is shown in Figure 6(b). We also analyzed the status of nuclear translocation of NF-*κ*B through immunofluorescence (Figure 6(c)). We found that allicin treatment effectively blocked the nuclear translocation of NF-*κ*B.

### 3.8. Allicin Inhibits the DSS-Induced Activation of STAT3

**Figure 7:**
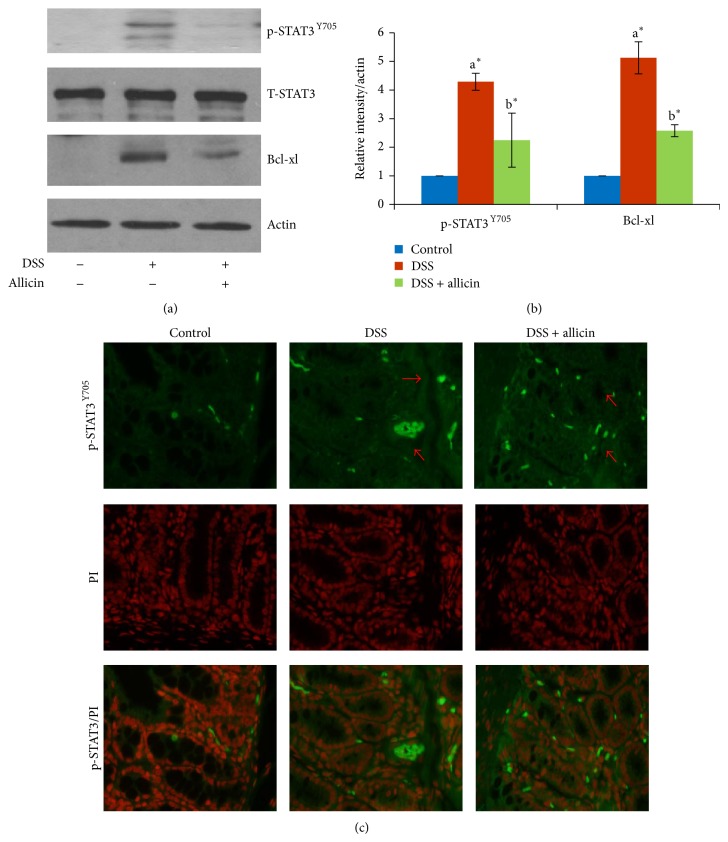
Allicin reduced the expression/activation of STAT3 and its downstream target Bcl-xl. (a) The DSS-induced animals showed increased expressions of p-STAT3^Y705^ and Bcl-xl (lane 2) compared with the control mice (lane 1). Allicin treatment (lane 3) decreased the expression of p-STAT3^Y705^ and Bcl-xl compared with the DSS-induced mice. The total STAT3 was unaltered in all of the experimental mice. (b) Quantification of the respective blots using the ImageJ software. (c) Immunofluorescence analysis of p-STAT3^Y705^. The values are expressed as the means ± S.D. In comparison of  ^a^control versus DSS,  ^b^DSS versus DSS + allicin. “∗” denotes a statistically significant difference at *P* < 0.05, and ns indicates a nonsignificant difference.

STAT3 is also known to be involved in colonic inflammation and activated by a variety of cytokines and growth factors [29]. Upon activation, STAT3 translocates to the nucleus, where it regulates genes involved in apoptosis (e.g., Bcl-xl), cell cycle progression (e.g., cyclin D1), migration, and survival depending on the cell type [30]. In our study, we assessed the expression of p-STAT3^Y705^, T-STAT3, and Bcl-xl in the control and experimental mice by immunoblot analysis. The expression of p-STAT3^Y705^ was increased in the mice administered DSS. However, allicin treatment inhibited the phosphorylation of STAT3 at Tyr705 (Figures 7(a) and 7(b)) and the translocation of p-STAT3^Y705^ into the nucleus (Figure 7(c)).

## 4. Discussion

Chronic inflammation leads to genomic instability, cell proliferation, and evasion from apoptosis and ultimately contributes to carcinogenesis [31]. Therefore, targeting abnormally overactive inflammation signaling is considered a crucial chemopreventive strategy. Numerous chemopreventive drugs have been found to modulate key molecules or events involved in inflammation-associated carcinogenic pathways. Numerous substances present in plant-based diets have been reported to modulate intracellular signal transduction pathways that often become awry during carcinogenesis.

Oxidative stress is one of the most crucial factors causing UC. Oxidative stress is known to damage cellular macromolecules such as DNA, lipids, and proteins. MDA is considered as a byproduct of lipid peroxidation known to be increased in UC [27, 32]. We noted that the level of MDA (Figure 5(a)) was increased in DSS-induced mice. But administration of allicin reduced the level of MDA in DSS-induced mice. In the present study, the activity of enzymic antioxidants such as SOD, CAT, GR, and GPx was decreased in DSS-induced mice. In general, ROS are known to neutralize the endogenous antioxidant enzymes. SOD converts O_2_^•−^ to H_2_O_2_, which is subsequently neutralized to water by CAT and GPx [33, 34]. In our findings, administration of allicin significantly increased the activity of enzymic antioxidants (Figure 5). Previous report shows that allicin is known to have stimulating effect on SOD and other antioxidant enzymes [13]. These findings indicated that allicin treatment of colitis may be reducing the extent of colonic injury by its antioxidant effect.

MPO is an index of neutrophil recruitment in the murine UC model. Therefore, the MPO activity may reflect more specific inflammatory events compared with cytokine concentrations. In our study, the colonic MPO activity was significantly increased in the DSS group compared with the noncolitic mice, and the group supplemented with allicin showed significantly (*P* < 0.05) decreased MPO activity (Figure 3(b)). Zhang et al. [13] reported that allicin treatment prevents an increase in MPO activity in mice. Accordingly, allicin blocks neutrophil infiltration into injured tissues. Cytokines are considered crucial signals in the intestinal immune system, and immune cells, such as, T cells, dendritic cells, macrophages, and intestinal epithelial cells, are involved in the secretion of various cytokines that regulate the inflammatory response in UC. Previous studies have revealed elevated levels of cytokines, such as TNF-*α*, IFN-*γ*, IL-1*β*, IL-6, IL-17, and IL-21, in UC [4, 35, 36]. In our study, we detected increased mRNA levels of* TNF-α*,* IL-1β*,* IL-6*, and* IL-17* in DSS-induced mice (Figure 4). However, the oral administration of allicin resulted in decreased mRNA levels of* TNF-α*,* IL-1β*,* IL-6*, and* IL-17*. Allicin is known to decrease the levels of proinflammatory cytokines in different disease models [37, 38].

NF-*κ*B is considered one of the major transcription factors involved in proinflammatory gene regulation and is generally present in the cytoplasm as a heterodimer complex of p65/p50 subunits combined with the inhibitory protein I*κ*B. Inflammatory stimuli induce rapid degradation of I*κ*B, and subsequently, the free NF-*κ*B molecule translocates into the nucleus, binds to target DNA elements, and activates the transcription of genes that encode proteins involved in inflammatory responses [39, 40]. In addition, NF-*κ*B is thought to be vital in the activation and progression of IBD in humans and of colitis in animals [41, 42]. Indeed, disease activity in mice with colitis is inhibited by antisense oligonucleotides that inhibit the p65 subunit of NF-*κ*B, which suggests a critical role for NF-*κ*B in mediating the inflammatory response [43]. In the present study, we showed increased expression of NF-*κ*B and decreased expression of I*κ*B in DSS-induced animals compared with the control mice (Figure 6). Attempts to control mucosal inflammation through the use of agents that block the NF-*κ*B pathway have achieved some success in murine models [5, 44]. Similarly, allicin treatment has been shown to block the activation of NF-*κ*B by activating I*κ*B [37, 45].

As a central downstream component of IL-6 signaling, STAT3 plays a vital role in the pathogenesis of IBD and is a cytokine-activated essential regulator in Th17 development [37]. The effect of STAT3 in intestinal inflammation is supported by the fact that activated STAT3 has been found in human IBD and animal colitis models [46, 47]. Another study showed that STAT3 is constitutively activated in CD patients compared with healthy individuals, and other STAT proteins are not constitutively activated [48]. Hence, the involvement of IL-6/STAT3 signaling in the pathogenesis of UC is well known [49]. Based on these findings, we strongly believed that controlling STAT3 expression during UC is a novel approach for avoiding the development of colitis-associated cancer [7, 28, 50]. Consistent with previous studies, we observed an increased expression of p-STAT3^Y705^ in DSS-induced mice (Figure 7(a)). Many natural products have been shown to inhibit STAT3 expression, leading to alleviation of the aggression of UC [51]. The results presented in this paper indicate that a significant beneficial effect of allicin is associated with reduced STAT3 activation in UC-induced mice.

In conclusion, the present findings suggest that allicin is an effective inhibitor of DSS-induced colitis in mice. The administration of allicin to mice treated with DSS attenuated acute inflammation in the colon. Therefore, allicin may reverse the precancerous state induced by DSS.

## Figures and Tables

**Table 1:** List of primers used in the study.

Number	Gene	Primer sequence	Primer length
1	TNF-*α*	Forward-5′-TGGTGACCAGGCTGTCGCTACA-3′	20
		Reverse-5′-TACAGTCACGGCTCCCGTGGG-3′	20

2	IL-1*β*	Forward-5′-TAGACAACTGCACTACAGGCTCCGA-3′	25
		Reverse-5′-GGGTCCGACAGCACGAGGCT-3′	20

3	IL-6	Forward-5′-ATGCTGGTGACAACCACGGCC-3′	21
		Reverse-5′-CCTCTGTGAAGTCTCCTCTCCGGAC-3′	25

4	IL-17	Forward-5′-CGTGGCCTCGATTGTCCGCC-3′	20
		Reverse-5′-GGTTTCTTAGGGGTCAGCCGCG-3′	22

5	*β*-actin	Forward-5′-GGCGGACTGTTACTGAGCTG-3′	20
		Reverse-5′-CTGCGCAAGTTAGGTTTTGTCA-3′	22
